# Structure, mechanism and inhibition of anthranilate phosphoribosyltransferase

**DOI:** 10.1098/rstb.2022.0039

**Published:** 2023-02-27

**Authors:** Thomas W. Scully, Wanting Jiao, Gerd Mittelstädt, Emily J. Parker

**Affiliations:** ^1^ Ferrier Research Institute, Victoria University of Wellington, Wellington 6140, New Zealand; ^2^ Maurice Wilkins Centre for Molecular Biodiscovery, Auckland 1142, New Zealand

**Keywords:** PRT, AnPRT, TrpD, tryptophan biosynthesis

## Abstract

Anthranilate phosphoribosyltransferase catalyses the second reaction in the biosynthesis of tryptophan from chorismate in microorganisms and plants. The enzyme is homodimeric with the active site located in the hinge region between two domains. A range of structures in complex with the substrates, substrate analogues and inhibitors have been determined, and these have provided insights into the catalytic mechanism of this enzyme. Substrate 5-phospho-d-ribose 1-diphosphate (PRPP) binds to the C-terminal domain and coordinates to Mg^2+^, in a site completed by two flexible loops. Binding of the second substrate anthranilate is more complex, featuring multiple binding sites along an anthranilate channel. This multi-modal binding is consistent with the substrate inhibition observed at high concentrations of anthranilate. A series of structures predict a dissociative mechanism for the reaction, similar to the reaction mechanisms elucidated for other phosphoribosyltransferases. As this enzyme is essential for some pathogens, efforts have been made to develop inhibitors for this enzyme. To date, the best inhibitors exploit the multiple binding sites for anthranilate.

This article is part of the theme issue ‘Reactivity and mechanism in chemical and synthetic biology’.

## 1. Introduction

Anthranilate phosphoribosyltransferase (AnPRT) catalyses the second committed step in tryptophan biosynthesis, in which a phosphoribosyl group is transferred from 5-phospho-d-ribose 1-diphosphate (PRPP) to anthranilate, forming *N*-(5-phosphoribosyl)-anthranilate (PRA) [1,2]. Tryptophan biosynthesis operates in plants and microorganisms [3–5]. Its absence in humans means that the enzymes of tryptophan biosynthesis are an attractive target for developing new therapeutics to combat infectious diseases [6]. AnPRT from *Mycobacterium tuberculosis* (*Mtu*AnPRT) has been validated as an antimicrobial drug target against *M. tuberculosis*, the pathogen responsible for tuberculosis [7–12]. Tuberculosis continues to cause several million deaths annually and the demand for new treatments for this disease will only increase as drug-resistant strains emerge [11–14].

**Figure 1.**
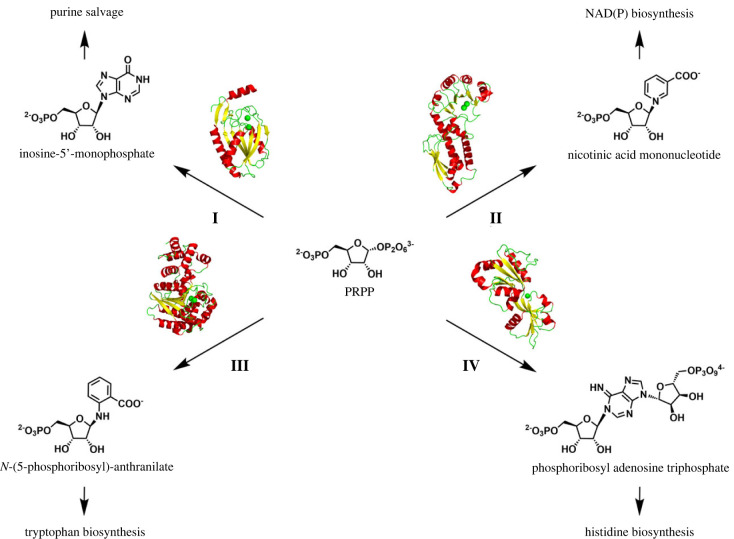
The PRT family. Overview of the architectures and metabolic role of PRT enzymes type I–IV. I: type I on the example of hypoxanthine PRT from *Trypanosoma cruzi* [16] (Protein Data Bank (PDB) ascension code: 1TC2). II: type II on the example of quinolinate PRT from *M. tuberculosis* [17] (PDB ascension code: 1QPR). III: type III on the example of AnPRT from *M. tuberculosis* [18] (PDB ascension code: 1ZVW). IV: type IV on the example of ATP-PRT from *Lactococcus lactis* [19] (PDB ascension code: 1Z7N). Representative monomeric structures are displayed as cartoon coloured according to secondary structure elements, helices (red), strands (yellow) and loops (green). The metal ions (green spheres) indicate the active site location. (Online version in colour.)

The enzyme AnPRT belongs to a wider group of phosphoribosyltransferases (PRTs) that all catalyse the transfer of a phosphoribosyl moiety from PRPP to a nitrogen-based nucleophile, including purines, pyrimidines and their biosynthetic precursors [15]. PRTs have been divided into four types with characteristic structural properties shared among members in each type (figure 1).

Type I PRT is the largest group and comprises mainly enzymes involved in the nucleotide salvage pathways [4,20–25]. This type includes the enzymes glutamine PRPP amidotransferase, PRPP synthase, uracil PRT, xanthine/hypoxanthine PRT, orotate PRT, guanine PRT and adenine PRT. Type I PRTs share a common *α*/*β* fold decorated by a highly flexible and multifunctional loop (*β*2-*β*3 loop) and a variable hood domain that play essential roles in catalysis and regulation of the corresponding PRT [15]. Type II PRTs include quinolinate and nicotinic acid PRTs of the nicotinamide pathway, and consist of an unusual open seven-stranded *α*/*β* barrel domain containing the active site in its centre and an N-terminal *α*/*β* plait domain that serves as a cap for the barrel of the neighbouring enzyme subunit [26–31]. Type IV PRTs are solely exemplified by adenosine triphosphate-PRT (ATP-PRT), which catalyses the first step of the histidine biosynthesis. ATP-PRTs share a common catalytic core, consisting of two interconnected *α*/*β* sandwich domains that contain the active site in the large cleft between them, but display two very diverse forms recruiting their own allosteric machinery [32,33]. The focus of this review is AnPRT, which is the only member of PRT type III.

## 2. Structure of anthranilate phosphoribosyltransferase

**Figure 2.**
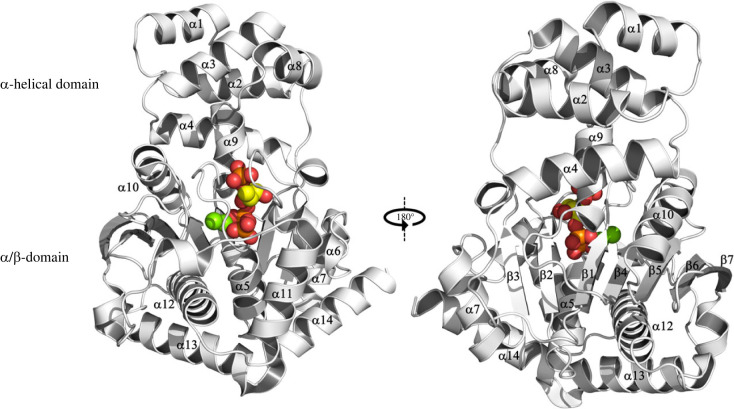
AnPRT fold. Three-dimensional structure of AnPRT on the example of PRPP-bound *Mtu*AnPRT (PDB ascension code: 1ZVW) [18] in cartoon representation with secondary structure elements labelled. Ligands PRPP (yellow) and Mg^2+^ (green) are displayed as spheres highlighting the active site location. Heteroatoms are coloured as follows: oxygen (red), phosphorous (orange). (Online version in colour.)

The structures of AnPRT from several organisms, across three domains of life, have been elucidated by X-ray crystallography over the past 20 years [2,18,34–37]. The overall structure of AnPRT is highly conserved (1.2–1.7 Å average root mean square deviation between monomeric units of different species) and unique among the PRT family, which led to its classification as PRT type III [2,34]. The AnPRT monomeric unit is between 330 and 380 amino acids in size and has two distinct domains, an N-terminal α-helical domain and a much larger C-terminal *α*/*β* domain, with the active site located in a cleft between the two domains (figure 2). The N-terminal domain is made up from a classical four-helix bundle [38] (*α*1–*α*4) augmented by helices *α*8 and *α*9. The AnPRT *α*/*β* domain is characterized by a large central mixed beta sheet (strands *β*1–*β*5 parallel, *β*6 and *β*7 antiparallel) and eight surrounding helices (*α*5–*α*7, *α*10–*α*14) as well as three helical motifs, one just after *β*3 and two in the long *β*7-*α*11 loop [2,18]. Occasionally the sequence is extended C-terminally to form another helix (*α*15), as observed for *Saccharolobus solfataricus* AnPRT (*Sso*AnPRT) [2].

All AnPRT structures determined to date are homodimeric, with their dimer interface being formed by interactions among helices *α*1, *α*3 and *α*8 of the N-terminal domain [18]. The interface is stabilized predominantly by backbone and hydrophobic interactions and the buried interface varies between individual enzymes from 750 to 1200 Å^2^. Because of this, the AnPRT dimer adopts an elongated structure (110 Å long versus 40 Å across), with the two active sites void of direct inter-dimer interactions [34,36]. Mutation studies carried out in *Sso*AnPRT have revealed that dimerization is important for the stability of AnPRT but does not play a direct role in catalytic activity [39].

There are few known homologues to the characteristic AnPRT fold. The enzymes of the nucleotide phosphorylase type II are highly similar to the AnPRT monomer with the C-terminal addition of an *α*/*β* hammerhead domain. The recently identified TrpD2 proteins are structural homologues to AnPRT but lack the strictly conserved PRPP binding motif and instead possess DNA-binding functionality. These homologues show a low, less than 20%, sequence identity to AnPRT [40]. Furthermore, a third homologous group of proteins of yet unknown function that is composed of the *α*/*β* domain alone can be found in higher plants [41].

In addition to the common monofunctional AnPRTs, a fused polypeptide (TrpGD) combining the two enzymes glutamine amido transferase and AnPRT exists. This architecture can be found predominantly in Enterobacteriaceae where it forms component II of the anthranilate synthase complex [42,43].

## 3. The kinetic and reaction mechanisms of anthranilate phosphoribosyltransferase

AnPRT catalyses the straightforward displacement of pyrophosphate from the anomeric carbon of PRPP by anthranilate to form PRA. The reaction is thought to occur with high stereoselectivity, although epimerization in solution means both anomers are observed for PRA. The reaction requires the presence of a divalent metal ion in the active site, which plays a role in activating the leaving pyrophosphate group in the AnPRT reaction. Most AnPRT enzymes characterized to date use Mg^2+^ ions, but preference for a Zn^2+^ ion for maximum activity has been reported for *Thermococcus kodakarensis* AnPRT (*Tko*AnPRT) [37].

Kinetics studies on *Escherichia coli* AnPRT [44], *Salmonella tryphimurium* AnPRT [45], *Mtu*AnPRT [46], *Sso*AnPRT [47,48] and *Saccharomyces cerevisiae* AnPRT (*Sce*AnPRT) [49] have revealed a general sequential mechanism for AnPRT enzymes. Crystal structures of *Mtu*AnPRT in the presence of substrates have shown that PRPP binding is required to fully form the catalytic binding site for anthranilate, suggesting an ordered sequential mechanism where PRPP binds first followed by anthranilate binding in the catalytic position [46]. By contrast, a random sequential mechanism has been proposed for *Sso*AnPRT based on structural evidence that anthranilate could bind in the catalytic position without the presence of PRPP [47]. Furthermore, substrate inhibition by anthranilate has been reported for *Mtu*AnPRT (*K*_i_ 45 ± 6 µM) [46] and *Tko*AnPRT (inhibition observed for anthranilate concentration above 4 µM) [37].

**Figure 3.**
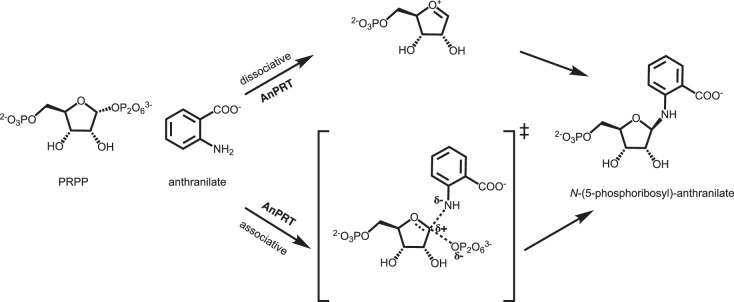
Proposed reaction mechanisms for AnPRT catalysed reaction, occurring through either a dissociative (top) or an associative (bottom) mechanism.

Two reaction mechanisms, namely dissociative or associative, have been proposed for AnPRT (figure 3). In a dissociative mechanism, bond breaking precedes bond making, and the pyrophosphate group of PRPP fully dissociates to form an oxocarbenium ion before nucleophilic attack by anthranilate. By contrast, in an associative mechanism, the reaction passes through a transition state (TS) with partial bond order between anthranilate and the anomeric carbon as well as between the anomeric carbon and the pyrophosphate group. Both dissociative and associative mechanisms have been described for the wider PRT family (figure 3). Kinetic isotope effect (KIE) studies and structural insights for some type I (orotate and hypoxanthine-guanine PRTs) and type IV PRTs (ATP-PRT) support a dissociative mechanism with a fully formed oxocarbenium ion [4,22,33,50–52]. By contrast, an associative mechanism has been proposed for the type II PRT enzyme, human nicotinamide PRT, based on structural evidence [31]. Although the general kinetic mechanisms are known for AnPRT, many of the precise details of catalysis remain unclear owing to the lack of any KIE studies on this enzyme to date. Nevertheless, kinetic studies and structural insights obtained for *Mtu*AnPRT in the presence of anthranilate analogues imply a likely dissociative mechanism, as discussed in detail below [46].

## 4. Phosphoribosyl pyrophosphate binding and the associated active site loop rearrangements

**Figure 4.**
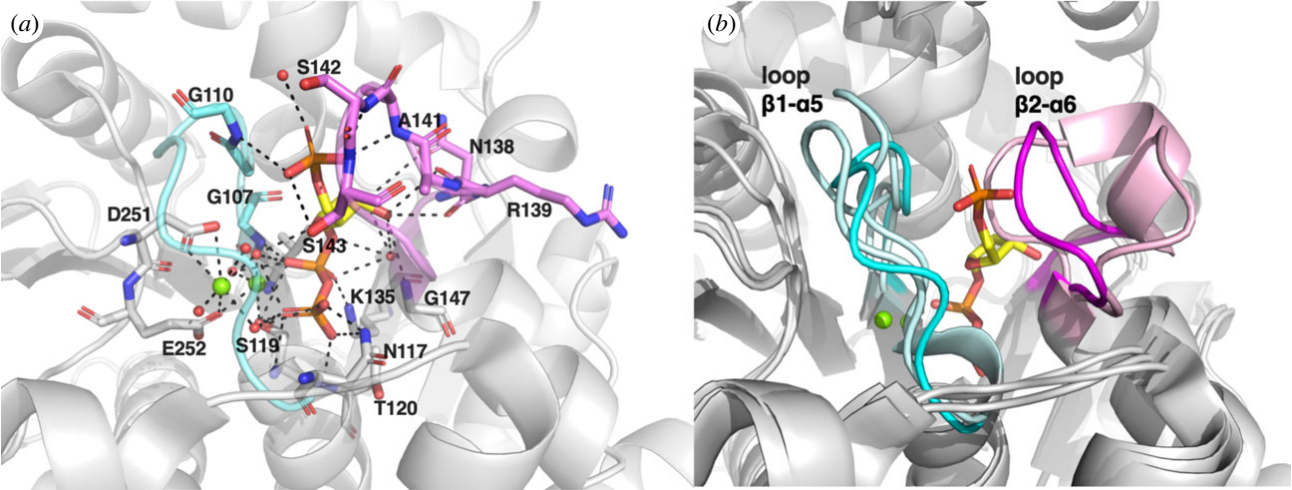
PRPP binding site and loop rearrangements associated with PRPP binding. (*a*) PRPP binding site in *Mtu*AnPRT (PDB 1ZVW [18]). PRPP is displayed with yellow carbon, Mg^2+^ ions are shown as green spheres, loop *β*1-α5 is coloured in cyan and loop *β*2-*α*6 is coloured in magenta. Polar interactions formed with PRPP and Mg^2+^ are displayed as black dashed lines. (*b*) Superimposition of apoensyme (PDB 2BPQ [18] and 3QR9 [6]) and PRPP-bound (PDB 1ZVW) *Mtu*AnPRT to highlight the conformational changes of loop *β*1-*α*5 (light cyan for apoenzyme structures and cyan for PRPP-bound structure) and *β*2-*α*6 (light pink for apoenzyme structures and magenta for PRPP-bound structure). (Online version in colour.)

Clear electron densities of PRPP have been observed in crystal structures of *Mtu*AnPRT (PDB 1ZVW) [18], *Sso*AnPRT (PDB 1ZXY) [47] and *Sce*AnPRT (PDB 7DSJ) [36]. PRPP adopts similar crystallographic conformations in *Mtu*AnPRT and *Sce*AnPRT, in which it binds deep in the C-terminal domain with enclosing loops *β*1-*α*5 and *β*2-*α*6 (figure 4*a*). The pyrophosphate group of PRPP binds the deepest and forms an extensive network of polar interactions with the N-terminal end of helix *α*5 (N117, T120 and S119 in *Mtu*AnPRT), helix *α*6 (G147 in *Mtu*AnPRT) and sheet *β*2 (K135 in *Mtu*AnPRT). In addition, the pyrophosphate group is coordinated to one Mg^2+^ ion (MG1), which is essential for catalysis. MG1 is also coordinated to a conserved Ser on helix *α*5 (S119 in *Mtu*AnPRT) and a conserved Glu on loop *β*5-*β*6 (E252 in *Mtu*AnPRT). A second Mg^2+^ ion (MG2) can be observed in most PRPP-bound structures of *Mtu*AnPRT, in which it shares a coordinated water molecule and the Glu residue with MG1 and is also coordinated to a conserved Asp on *β*5-*β*6 (D251 in *Mtu*AnPRT). The 5′-phosphate group interacts with the *β*1-*α*5 loop (G107 and G110 in *Mtu*AnPRT), which contains a conserved ‘GTGGD’ motif and the *β*2-*α*6 loop (S142 and S143 in *Mtu*AnPRT). The ribose moiety is held in position by polar interactions with the backbones of residues on the *β*2-*α*6 loop (e.g. N138-R139 in *Mtu*AnPRT and G141-K142 in *Sce*AnPRT).

PRPP was observed to bind in a different conformation in *Sso*AnPRT, in which one PRPP is coordinated to two Mg^2+^ ions via both its pyrophosphate and 5′-phosphate groups [18]. However, it was established that the catalytically active Mg^2+^ : PRPP complex is the singly coordinated PRPP, as observed in *Mtu*AnPRT and *Sce*AnPRT [53].

The flexible loops *β*1-*α*5 and *β*2-*α*6 undergo significant conformational rearrangements upon PRPP binding (figure 4*b*). In the substrate-free crystal structures of AnPRTs, these two loops are either observed in various conformations or disordered (e.g. *Pectobacterium carotovorum* AnPRT (*Pca*AnPRT) [34], *Sce*AnPRT [36], *Xanthomonas campestris* AnPRT (PDB 4HKM) and *Acinetobacter baylyi* AnPRT (PDB 4GTN)). While in the presence of PRPP, significant conformational rearrangements and ordering of these two loops can be observed in *Mtu*AnPRT [6,18] (figure 4*b*), *Pca*AnPRT [34] (PDB 1KDH and 1KGZ) and *Sce*AnPRT [36] (PDBs 7DSM and 7DSJ) to accommodate and contribute significant interactions to PRPP binding. This observed loop conformational rearrangement is essential for PRPP recognition and plays an important role in anthranilate binding by forming the catalytically competent binding site for anthranilate.

## 5. Anthranilate binding and the anthranilate channel

**Figure 5.**
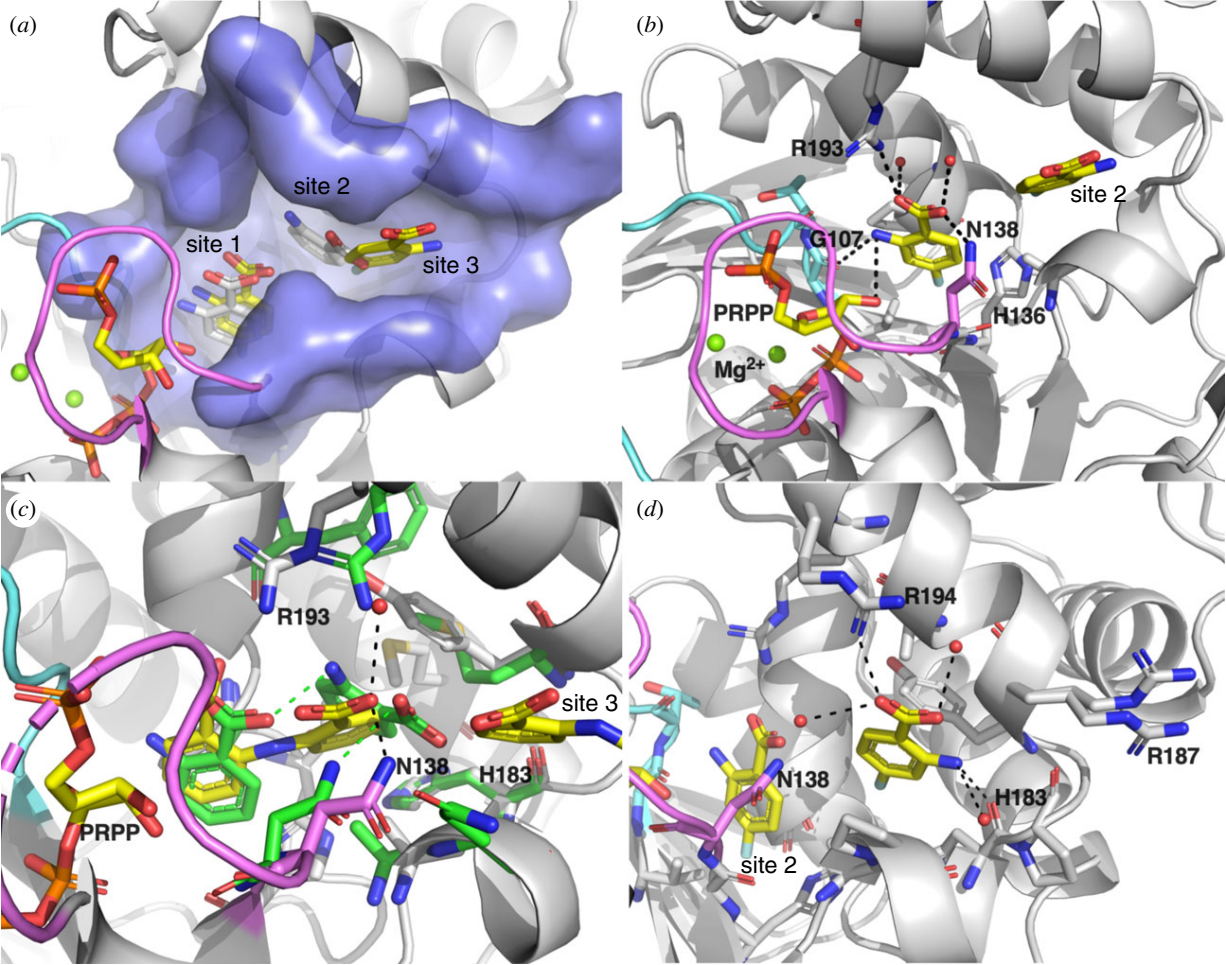
Anthranilate channel and anthranilate binding sites. Active site loops *β*1-*α*5 and *β*2-*α*6 are shown in light cyan and magenta, respectively. (*a*) Anthranilate channel in *Mtu*AnPRT (PDB 4N5V [46]) is displayed with a light blue surface. A PRPP molecule and 4-fluoroanthranilate molecules bound in site 1 and site 3 of *Mtu*AnPRT (PDB 4N5V) are shown with yellow carbon atoms. Anthranilate molecules bound in site 1 and site 2 in *Sso*AnPRT (PDB 1ZYK [47]) are shown with white carbon atoms. (*b*) Anthranilate binding site 1 in *Mtu*AnPRT (PDB 4N5V). Polar interactions are displayed as black dashed lines. (*c*) Anthranilate binding site 2. Superimposed active sites of *Mtu*AnPRT (PDB 3QQS [6]) and *Sso*AnPRT (green carbon atoms, PDB 2GVQ [47]). The bianthranilate-like inhibitor (ACS172) in *Mtu*AnPRT (yellow carbon atoms) occupies sites 1 and 2 simultaneously. Polar interactions formed by the site 2 anthranilate portion of ACS172 in *Mtu*AnPRT are displayed as black dashed lines, and those formed by site 2 anthranilate in *Sso*AnPRT are displayed as green dashed lines. (*d*) Anthranilate binding site 3 in *Mtu*AnPRT (PDB 4N5V). Polar interactions are displayed as black dashed lines. (Online version in colour.)

Multiple binding sites of anthranilate on AnPRT enzymes have been predicted by molecular modelling for *Mtu*AnPRT [18] and later observed experimentally in crystal structures of *Mtu*AnPRT with anthranilate analogues and various inhibitors bound [6,46] and in crystal structures of *Sso*AnPRT with anthranilate bound [47]. Three binding sites can be identified along an anthranilate channel, which were proposed to capture and shuttle anthranilate from solution to the catalytic site (figure 5*a*), and to account for the observation of substrate inhibition by anthranilate. Site 1 is the catalytically competent binding site for anthranilate and is in close proximity to PRPP. Anthranilate at site 1 is surrounded by residues from the closed loops *β*1-*α*5 and *β*2-*α*6, helices *α*8 and *α*9, and sheet *β*2 (figure 5*b*). The carboxylate group of anthranilate is held by the side chain of a conserved Arg on helix *α*8 (R193 in *Mtu*AnPRT) and forms polar interactions with loop *β*2-*α*6 (N138 in *Mtu*AnPRT). Amino acid substitutions at N139 and R193 of *Mtu*AnPRT caused moderate to a significant impairment to catalysis and a significant increase in the apparent *K*_m_ for anthranilate, confirming their importance in anthranilate binding and catalytic function [35]. The amino group of anthranilate forms polar interactions with the ribose moiety of PRPP and the backbone of a Gly from the conserved ‘GTGGD’ motif in loop *β*1-*α*5 (G107 in *Mtu*AnPRT).

Site 2 is a transient site between sites 1 and 3, surrounded largely by hydrophobic residues. Occupancy of site 2 has been observed in *Sso*AnPRT [47] (PDB 2GVQ) and *Mtu*AnPRT [6] (e.g. PDB 3QQS). Site 2 anthranilate forms very few polar interactions, displaying hydrogen bonds only to an Asn residue on loop *β*2-*α*6 and the anthranilate in site 1 (figure 5*c*).

Occupancy of site 3, the outermost site, has only been observed crystallographically in *Mtu*AnPRT [6,46]. By soaking PRPP, Mg^2+^ and anthranilate analogues for various time periods prior to crystal harvesting and freezing, a series of snapshots of catalysis and substrate binding was obtained. In those time course crystal soaking experiments, site 3 was found to be consistently the first site to be occupied by anthranilate analogues and was proposed to play a role in the capturing of anthranilate [46]. Anthranilate at site 3 of *Mtu*AnPRT interacts with R194, a non-conserved residue on helix *α*8 via its carboxylate group and the amino group forms a hydrogen bond with the backbone of H183 (figure 5*d*).

## 6. Proposed catalytic cycle for *Mtu*AnPRT

**Figure 6.**
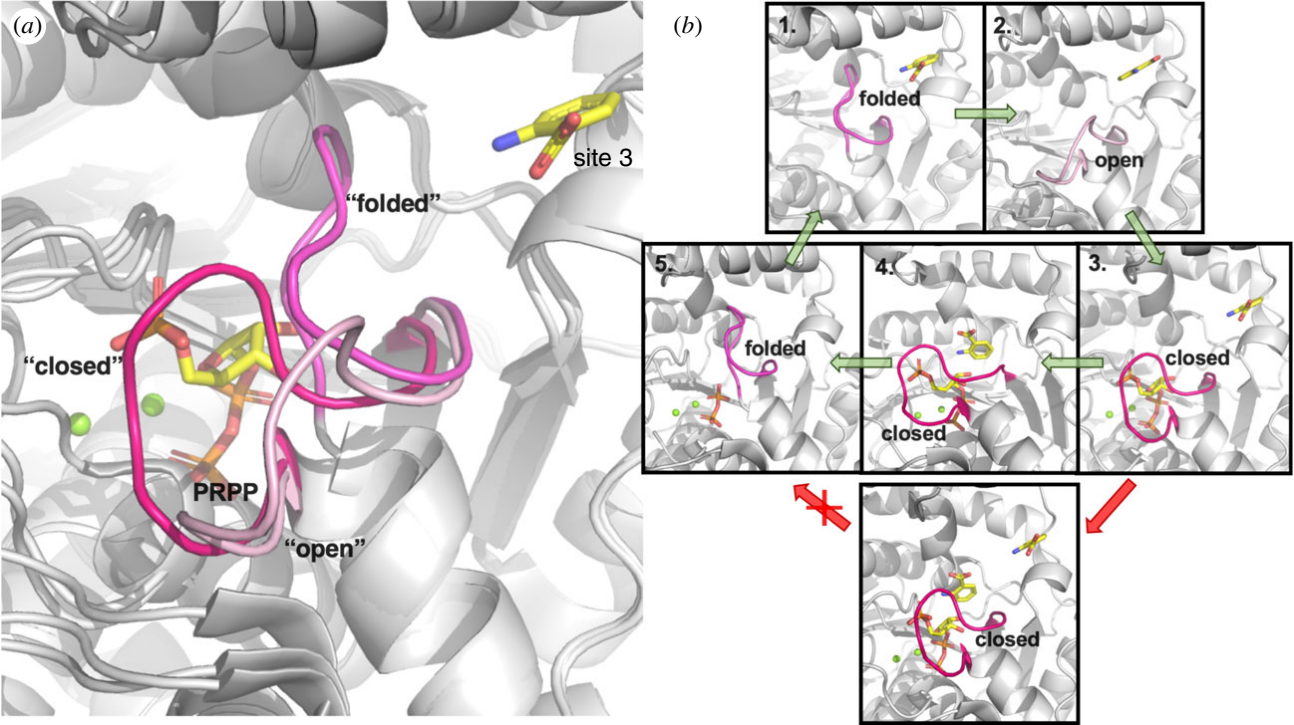
(*a*) The open (PDB 3QR9 [6]), closed (PDB 1ZVW [18]) and folded (PDB 4X5D [35]) conformations of loop *β*2-*α*6 in *Mtu*AnPRT (coloured in different shades of magenta). PRPP (from 1ZVW) and one anthranilate bound in site 3 (from 4X5D) are displayed with yellow carbon atoms. (*b*) Proposed ligand binding orders and active site loop movements for the catalytic cycle of *Mtu*AnPRT by Cookson *et al.* [35]. The conformations of loop *β*2-*α*6 are labelled as open, folded and closed accordingly. (Online version in colour.)

In addition to the open and closed conformations of loop *β*2-*α*6 observed in the substrate-free and PRPP-bound crystal structures of AnPRTs, another ‘folded’ conformation of this loop has been observed in crystal structures of wild-type and R193A variant of *Mtu*AnPRT when anthranilate or its analogue binds only at site 3 after a short period of soaking [35,46]. This distinct ‘folded’ conformation has loop *β*2-*α*6 parked in the anthranilate channel, blocking access to site 2 and the catalytic binding site (site 1) of anthranilate (figure 6*a*).

The large amount of structural information has allowed a mechanism to be proposed for *Mtu*AnPRT that is consistent with the known kinetic features (figure 6*b*) [6,9,18,46]. The proposed catalytic cycle begins with trapping anthranilate at site 3 with loop *β*2-*α*6 in a folded conformation (step 1, figure 6*b*). Loop *β*2-*α*6 then rearranges to the open conformation to allow PRPP entrance to the active site (step 2, figure 6*b*), which is followed by the closure of loop *β*2-*α*6 to stabilize PRPP binding and fully form the catalytic binding site for anthranilate (step 3, figure 6*b*). Anthranilate is then shuttled to site 1 and the reaction occurs (step 4, figure 6*b*). It has been proposed that the product PRA is released before pyrophosphate, which is deeply buried in the C-terminal domain. Loop *β*2-*α*6 moves away from the closed conformation to adopt the folded conformation to release the pyrophosphate from the active site (step 5, figure 6*b*). The active site conformation is then regenerated for the next catalytic cycle.

The proposed mechanism also explains the reported substrate inhibition of *Mtu*AnPRT by anthranilate [46]. In the presence of high concentration of anthranilate, it is likely that more than one anthranilate molecule will bind to *Mtu*AnPRT when loop *β*2-*α*6 is in the closed conformation and the anthranilate channel is not obstructed (figure 6*b*). In this case, the loop *β*2-*α*6 cannot move back into the folded conformation as the anthranilate channel is occupied, and the pyrophosphate is probably trapped in the active site, which disrupts the catalytic cycle. It should be noted that this mechanism is only proposed for the *Mtu*AnPRT enzyme. However, substrate inhibition by anthranilate has also been reported for *Tko*AnPRT [37], suggesting this could be a more common feature among AnPRT enzymes.

## 7. Inhibition of anthranilate phosphoribosyltransferase

The essential nature of tryptophan biosynthesis for some pathogens has created considerable interest in exploring inhibition of AnPRT. In particular, it has been shown that the tryptophan biosynthetic pathway genes are among those that enable *M. tuberculosis* to overcome the innate host immune defence [7]. In addition, the use of inhibitors and alternative substrates for AnPRT has helped to elucidate significant structural information around substrate binding, active site architecture and reaction mechanism for AnPRT as described above.

Several anthranilate-like compounds have been screened as inhibitors for *Mtu*AnPRT [6,9,46,53,54]. The binding modes for these molecules highlight the multiple binding sites for anthranilate and illuminate the role of the anthranilate channel in AnPRT catalysis. The best hit from the initial screen was the bianthranilate-like 2-(2-carboxyphenylamino)benzoate (ACS172), which is a competitive inhibitor with respect to anthranilate (IC_50_ 40 ± 2 µM [9]). The crystal structure of *Mtu*AnPRT complexed with ACS172 reveals two distinct modes which overlap with the multiple anthranilate binding sites observed for the enzyme [6]. Kinetic and crystallographic data indicate that this inhibitor binds to the anthranilate channel and prevents the binding of anthranilate and its migration to the catalytic site [9]. However, ACS172 resulted only in partial inhibition of the enzymatic activity, even at high concentrations, indicating that it may not be accessing the catalytic anthranilate binding site [9]. This compound was shown to reduce cell growth of *Mycobacterium marinum* at concentrations between 25 and 600 µg ml^−1^ [9]. *Mycobacterium marinum* causes disease in fishes and responds to anti-TB drugs in a similar way to *M. tuberculosis*.

**Figure 7.**
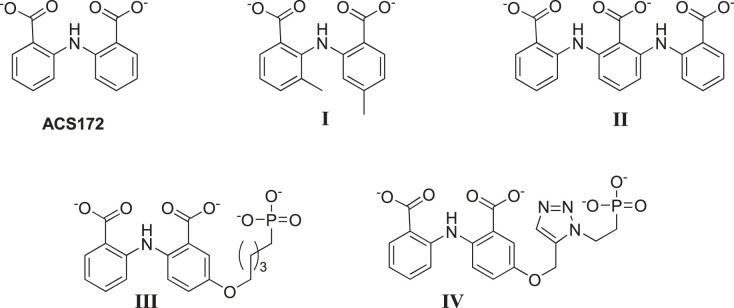
Structures of anthranilate-like inhibitors ACS172, 2-((2-carboxy-5-methylphenyl)amino) (**I**) and 2,6-bis((2-carboxy-5-methylphenyl)amino)-3-methylbenzoate (**II**) [9], as well as phosphonates 2-(2-carboxyphenylamino)-5-(5-phosphonopentyloxy)benzoic acid (**III**) and 2-(2-carboxyphenylamino)-5-((1-(2-phosphonoethyl)-1*H*-1,2,3-triazol-4-yl)methoxy)benzoic acid (**IV**) [53].

The inhibition results for the bianthranilate compound ACS172 encouraged the synthesis and testing of a further set of analogues based on this chemical scaffold. While replacement of the secondary amine or one of the carboxylate functionalities reduced inhibition, the inclusion of various substituents on one or both of the phenyl moieties increased efficacy owing to stabilizing interactions with residues of anthranilate binding sites 2 and 3. One of these 2-((2-carboxy-5-methylphenyl)amino)-3-methylbenzoate (**I**) caused complete inhibition of the enzyme and was shown to bind into the catalytically relevant anthranilate binding site (IC_50_ = 6.8 ± 0.3 µM) [9]. In addition, an extended trianthranilate-like compound, 2,6-bis((2-carboxyphenyl)amino)benzoate (**II**), was reported to be a 40-fold more potent inhibitor for *Mtu*AnPRT than the original ACS172 (IC_50_ = 2.2 ± 0.1 µM, figure 7) [9].

Attempts were also made to extend binding of the bianthranilate-like inhibitor ACS172 into the PRPP binding site, and a series of phosphonate-containing compounds was synthesized (**III**, **IV**). While some of these inhibitors showed a modest increase in potency relative to ASC172 (IC_50_ = 16 ± 2 µM and IC_50_ = 11 ± 1 µM for **III** and **IV**, respectively), crystallographic and kinetic data suggested the flexible phosphonate-containing tails were not accessing the PRPP binding sites. As this site is well defined, it may be that more-rigid phosphoribosyl-mimicking components need to be incorporated [53].

The dissociative mechanism predicted for AnPRT parallels the catalytic mechanisms of other PRPP-using PRT enzymes, and this feature has been exploited in the design of powerful inhibitors for these enzymes [51]. For example, detailed KIE analysis and TS modelling for *Plasmodium falciparum* hypoxanthine-guanine-xanthine PRT is consistent with a dissociative reaction with an asymmetric TS with low bond order to both the departing diphosphate group and incoming N from hypoxanthine, with substantial positive charge build up on the anomeric carbon [55]. This mechanism accounts for the powerful inhibition observed for iminoribitols immucillinHP and immucillinGP [56].

## 8. Conclusion and outlook

The transfer of the phosphoribosyl group from PRPP catalysed by the phosphoribosyltransferases is an important reaction in metabolism. Elegant and detailed mechanistic studies have defined the reaction coordinate and provided information to guide specific and potent inhibitor design. While detailed KIE measurements and mechanistic studies are yet to be performed for AnPRT, structural information from many ligand-bound structures, particularly for *Mtu*AnPRT, has illuminated many catalytic details and supports a dissociative mechanism for this enzyme. In particular, the observation of multiple binding sites for the substrate anthranilate in at least some AnPRT homologues may lead to specific, tailored inhibitors for this enzyme, when combined with detailed mechanistic information. The essential nature of tryptophan biosynthesis in some pathogens has meant there continues to be interest in the development of inhibitors for this enzyme.

## Data Availability

This article has no additional data.
